# Impact of surveillance of hospital-acquired infections on the incidence of ventilator-associated pneumonia in intensive care units: a quasi-experimental study

**DOI:** 10.1186/cc11484

**Published:** 2012-08-21

**Authors:** Thomas Bénet, Bernard Allaouchiche, Laurent Argaud, Philippe Vanhems

**Affiliations:** 1 Infection Control and Epidemiology Unit, Edouard Herriot Hospital, Hospices Civils de Lyon, Lyon, France; 2 Epidemiology and Public Health Group, CNRS UMR 5558, University of Lyon 1, University of Lyon, Lyon, France; 3 Intensive Care Unit, Edouard Herriot Hospital, Hospices Civils de Lyon, Lyon, France; 4 Medical Intensive Care Unit, Hospices Civils de Lyon, Edouard Herriot Hospital, Lyon, France

## Abstract

**Introduction:**

The preventive impact of hospital-acquired infection (HAI) surveillance is difficult to assess. Our objective was to investigate the effect of HAI surveillance disruption on ventilator-associated pneumonia (VAP) incidence.

**Methods:**

A quasi-experimental study with an intervention group and a control group was conducted between 1 January 2004 and 31 December 2010 in two intensive care units (ICUs) of a university hospital that participated in a national HAI surveillance network. Surveillance was interrupted during the year 2007 in unit A (intervention group) and was continuous in unit B (control group). Period 1 (pre-test period) comprised patients hospitalized during 2004 to 2006, and period 2 (post-test period) involved patients hospitalized during 2008 to 2010. Patients hospitalized ≥48 hours and intubated during their stay were included. Multivariate Poisson regression was fitted to ascertain the influence of surveillance disruption.

**Results:**

A total of 2,771 patients, accounting for 19,848 intubation-days at risk, were studied; 307 had VAP. The VAP attack rate increased in unit A from 7.8% during period 1 to 17.1% during period 2 (*P *<0.001); in unit B, it was 7.2% and 11.2% for the two periods respectively (*P *= 0.17). Adjusted VAP incidence rose in unit A after surveillance disruption (incidence rate ratio = 2.17, 95% confidence interval 1.05 to 4.47, *P *= 0.036), independently of VAP trend; no change was observed in unit B. All-cause mortality and length of stay increased (*P *= 0.028 and *P *= 0.038, respectively) in unit A between periods 1 and 2. In unit B, no change in mortality was observed (*P *= 0.22), while length of stay decreased between periods 1 and 2 (*P *= 0.002).

**Conclusions:**

VAP incidence, length of stay and all-cause mortality rose after HAI surveillance disruption in ICU, which suggests a specific effect of HAI surveillance on VAP prevention and reinforces the role of data feedback and counselling as a mechanism to facilitate performance improvement.

## Introduction

Ventilator-associated pneumonia (VAP) is a major concern in intensive care units (ICUs) because of its high incidence and related mortality [1]. In the French national surveillance system of hospital-acquired infections (HAIs) in ICUs, 12.4% of intubated patients in 2010 developed VAP during hospital stay, the most frequent HAI site in ICU populations [2]. Mortality attributable to VAP has been reported to be as high as 40 to 50% [1], with a recent cohort study estimating a two-month attributable mortality of 5.9% [3]. It has been postulated that at least 20% of HAIs could be prevented with the implementation of appropriate measures [4-6], as several investigations with multifaceted interventions have noted decreases up to 70% in VAP rates [7-9]. HAI surveillance might have a protective effect against HAI occurrence. However, the strength and causality of the association between surveillance and HAI incidence reduction are difficult to estimate.

Several studies have demonstrated decreased HAI incidence shortly after the implementation of nation-wide surveillance [10-15]. For example, the Krankenhaus Infektions Surveillance System in Germany reported a 20% decline of nosocomial pneumonia incidence between the first and third years [11,16]. However, HAI rates at implementation of surveillance were high [10-12]. Also, the diminution of incidence could be explained in part by regression to the mean [17]. Evaluation did not include a control group. The decrease of HAI in these studies could be ascribed to confounding factors, such as disease severity or modification of care practices. Moreover, it has been underlined that investigations into VAP prevention should present benefits in patient outcome (mortality and duration of stay) in addition to reduction of VAP incidence [18].

We hypothesize a converse relationship compared to that suggested previously, namely, disruption of HAI surveillance might induce an increase in HAI rates. It is difficult to perform controlled randomized trials to confirm or refute this premise because of logistic reasons and since HAI rates in units without surveillance are unknown. A quasi-experimental design permitted us to test the above supposition [17,19,20]. A unit with continuous surveillance served as a control group, while a unit with surveillance disruption was the intervention group. Therefore, the study objective was to assess the effect of HAI surveillance disruption on VAP incidence in ICUs with a quasi-experimental design.

## Materials and methods

### Study setting and intervention

A quasi-experimental study with intervention and control groups was undertaken between 2004 and 2010 in two ICUs of Edouard Herriot Hospital in Lyon, France (units A and B, respectively). Unit A is a medical/surgical polyvalent ICU which contains 12 single rooms. Unit B is a medical polyvalent ICU with 15 single rooms. The two units did not differ in their staff/bed ratio, but both conformed to French law. These ICUs have participated in a national HAI surveillance network since 1999, according to a programme described elsewhere [15,21,22]. Surveillance is based on prospective collection of HAIs in units over the years that permits incidence calculation. A nurse or physician of the infection control unit of our hospital is regularly present in each unit for surveillance and to discuss prevention practices with ICU staff. Feedback is provided annually to ICU physicians and nurses by the infection control team, and a summary of the results is sent each semester to heads of both units. During surveillance periods, similar expertise and counselling were imparted in each unit by the infection control staff.

Usually, surveillance is continuous and conducted each year. In unit A, surveillance was interrupted because of infection control team re-organisation in 2007 but was continuous in 2004 to 2006 and 2008 to 2010. Also, the combination of the frequent physical presence of infection control staff for surveillance and data feedback was stopped in unit A in 2007. In unit B, surveillance was not interrupted between 2004 and 2010. The intervention was HAI surveillance disruption in unit A in 2007. In other words, the intervention group was unit A with HAI surveillance disruption in 2007, and the control group was unit B with no HAI surveillance disruption in 2007. The pre-test period (period 1) included patients discharged between 1 January 2004 and 31 December 2006, and the post-test period (period 2) comprised patients discharged between 1 January 2008 and 31 December 2010.

No other specific intervention in VAP prevention was implemented selectively in unit A or unit B between periods 1 and 2. Both units followed the same recommendations.

### Study population

All patients hospitalised ≥48 hours in the ICUs were entered in the surveillance programme until their discharge. Data were collected prospectively over the years by infection control nurses or physicians on a standardised form and incorporated demographic characteristics, underlying disease severity, risk factors for HAIs, exposure to mechanical ventilation, date and site of infection, etiological agents and patient outcome. All patients, hospitalized in ICUs ≥48 hours and intubated or tracheotomised during ICU stay, were analysed.

### Definitions of VAP

VAP [23] was defined according to the following criteria [24]:

- Chest X-rays exhibiting lung infiltrates;

- Temperature >38°C or leukocyte count >12,000/mm^3 ^or <4,000/mm^3^;

- One of the following: 1) sputum modification, 2) suggestive auscultation, 3) low oxyhaemoglobin saturation, or 4) increased pulmonary oxygen consumption;

- And one of the following: 1) directed broncho-alveolar lavage (BAL)-positive culture at a threshold of 10^4 ^cfu/ml in BAL or 10^3 ^cfu/ml in mini-BAL [25], 2) fibreoptic bronchoscopy specimen-positive culture at a threshold of 10^6 ^cfu/ml, or 3) one of the following: positive pleural or blood cultures without any other site of infection, pulmonary or pleural abscess, histopathological evidence of pneumonia or cultures positive for specific agents. In patients presenting consecutive VAP episodes, only the first episode was considered.

### Statistical analysis

Firstly, descriptive analysis was performed. Quantitative variables were reported as number and percentage, and qualitative variables, as mean + standard deviation (SD). The VAP "attack rate" was the number of VAPs per 100 intubated patients, and VAP incidence was the number of VAPs per 1,000 intubation-days at risk.

Secondly, analysis was conducted with Poisson regression to assess temporal trends and the effect of surveillance disruption [26]. Bivariate Poisson regression was fitted with number of VAPs as dependent variable, time (per quarter) and HAI surveillance disruption in 2007 as independent variables and the number of intubation-days at risk as offset. The following potential confounding factors were tested, first by univariate analysis: gender, age, simplified acute physiological score II (SAPSII) at admission, reason for admission (medical, surgery), immunosuppression, and antibiotics received at admission. Backward step-wise multivariate analysis followed. Trends (per quarter) and HAI surveillance disruption in 2007 were forced into the model; potential confounding factors were entered initially if their *P-*value after univariate analysis was ≤0.15; models were compared by the log-likelihood-ratio test. A full model was fitted with two independent and all potential confounding factors. All statistical tests were two-tailed, and *P *<0.05 was considered as statistically significant. Missing data were avoided by complete case analysis.

Studies, such as this one, do not require ethics committee approval because they are based on an observational surveillance database approved under national regulations (*Comité National Informatique et Liberté*). According to French law, current surveillance of nosocomial infections in hospital and epidemiological observational surveys do not need institutional review board authorization or written consent.

## Results

### Study participants

Of the 4,411 patients included in the surveillance of both units during the study period, 2,915 (61.1%) were exposed to mechanical ventilation during their hospital stay. Data were missing for 4.9% (n = 144) of intubated patients and were, therefore, excluded.

Totally, 2,771 patients, accounting for 37,330 hospitalisation days, 26,768 intubation-days and 19,848 intubation-days at risk, were analysed. Among them, 940 (34%) were hospitalised in unit A, and 1,831 (66%) in unit B. Overall, 62% (n = 1,724) were men, and mean ± SD age was 60 ± 16 years. Totally, 61% (n = 1,694) received antibiotics at admission, 41% (n = 1,142) came from home, and 26% (n = 721) were immunosuppressed. Mean ± SD SAPSII score was 53 ± 20. All-cause in-hospital mortality was 26% (n = 712).

Overall, 307 patients had VAP. The VAP attack rate was 11.1 per 100 intubated patients and the VAP incidence rate was 15.5 (95% confidence interval (95% CI) 13.8 to 17.3) per 1,000 intubation-days at risk.

### Characteristics by group and period

Table 1 reports the characteristics of hospitalised patients by group and period. In the intervention group with surveillance interrupted in 2007, patients hospitalised during period 2 compared to period 1 more often came from home (*P *= 0.004), were more immunosuppressed (*P *<0.001), more often had surgery as diagnosis category (*P *<0.001), received antibiotics less frequently at admission (*P *<.001), were older (*P *= 0.022), and presented lower mean SAPSII (*P *<0.001). In the control group with continuous surveillance, patients hospitalised during period 2 compared to period 1 were less likely to come from home (*P *<0.001), were more immunosuppressed (*P *<0.001), and received antibiotics more frequently at admission (*P *<0.001).

**Table 1:** Description of the study population by period and ICU, Edouard Herriot Hospital, Lyon (France), 2004-2010.

Characteristics	Intervention group: Interrupted surveillance, unit A			Control group: Continuous surveillance, unit B		
	
	Period 1 2004 to 2006 (n = 448)	Period 2 2007 to 2010 (n = 492)	*P*	Period 1 2004 to 2006 (n = 743)	Period 2 2007 to 2010 (n = 881)	*P*
Categorical variable, n (%)						
Gender, male	308 (68.8)	320 (65.0)	0.23	439 (59.1)	531 (60.3)	0.63
Patient origin			0.004			<0.001
Home	70 (15.6)	114 (23.2)		421 (56.7)	421 (47.8)	
Other unit/hospital	378 (84.4)	378 (76.8)		322 (43.3)	460 (52.2)	
Immunosuppressed^a^	153 (34)	263 (53)	<0.001	98 (13.2)	175 (19.9)	<0.001
Diagnosis category^a^			<0.001			0.060
Medical	265 (59.2)	161 (32.7)		660 (88.8)	807 (91.6)	
Surgery	183 (40.9)	331 (67.3)		83 (11.2)	74 (8.4)	
Antibiotics^a^	284 (63.4)	230 (46.8)	<0.001	397 (53.4)	629 (71.4)	<0.001
Deceased in-hospital	60 (13.5)	91 (18.8)	0.028	236 (31.9)	256 (29.1)	0.22
						
Continuous variable, mean (SD)						
Age, years^a^	56.3 (14.9)	58.5 (15.5)	0.022	61.1 (16.4)	60.7 (59.6)	0.61
SAPSII^a^	51.7 (17.9)	45.4 (21.0)	<0.001	55.6 (19.8)	55.5 (18.8)	0.95
Length of hospital stay, days	12.2 (18.6)	15.3 (25.7)	0.038	14.8 (20.3)	12.0 (17.0)	0.0023
Length of invasive mechanical ventilation, days	7.7 (14.5)	11.3 (24.7)	0.007	11.1 (19.0)	8.5 (14.5)	0.0015
						
Incidence of VAP						
Number of VAP	35	84		68	99	
Attack rate^b^	7.8	17.1	<0.001	9.2	11.2	0.17
Incidence^c ^(95% CI)	13.4 (9.5-18.4)	22.9 (18.4-28.2)		11.2 (8.7-14.1)	17.1 (14.0-20.8)	

NOTE: CI, confidence interval; SD, standard deviation; SAPSII, simplified acute physiological score II; VAP, ventilator-associated pneumonia. ^a ^At ICU admission. ^b ^Number of VAP per 100 intubated patients. ^c ^Number of VAP per 1,000 intubation-days.

### Duration of exposure by group and period

In the intervention group with surveillance interrupted in 2007, mean length of ICU stay and of invasive mechanical ventilation was longer during period 2 compared to period 1 (*P *= 0.038 and *P *= 0.007, respectively). In the control group, the length of invasive mechanical ventilation was shorter during period 2 compared to period 1 (*P *= 0.0023).

In patients who had VAP, mean ± SD length of invasive mechanical ventilation before VAP onset did not change between periods 1 and 2 in the intervention group (11.6 ± 12.5 days vs. 14.0 ± 15.0 days, respectively, *P *= 0.4) and in the control group (13.1 ± 11.6 days vs. 10.4 ± 9.9 days, respectively, *P *= 0.11). In patients who did not suffer from VAP, no difference was found regarding mean ± SD length of hospital stay between periods 1 and 2 in the intervention group (9.3 ± 11.9 days vs. 9.9 ± 14.8 days, respectively, *P *= 0.5), whereas it decreased in the control group (11.3 ± 13.0 days vs. 9.1 ± 9.9 days respectively, *P *<0.001).

### Outcome

In the intervention group with surveillance interrupted in 2007, all-cause in-hospital mortality increased from 13.5% (n = 60) to 18.8% (n = 91) between periods 1 and 2 (*P *= 0.028). In the control group, all-cause in-hospital mortality was stable between the periods (*P *= 0.22).

### Effect of surveillance disruption on VAP incidence

**Figure 1:**
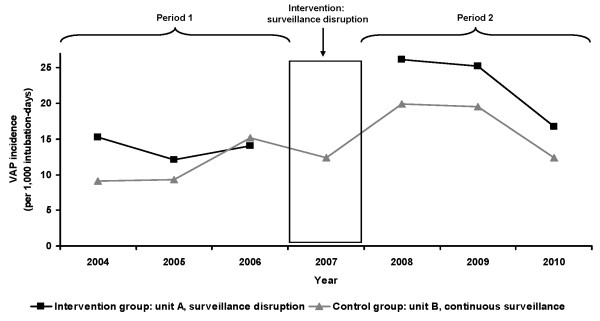
**Trend of ventilator-associated pneumonia incidence in ICU, Edouard Herriot Hospital, Lyon (France), 2004-2010**. NOTE: The intervention group was unit A with surveillance disruption in 2007, the control group was unit B with continuous surveillance. Period 1 (pre-test period) comprised patients hospitalized during 2004 to 2006, period 2 (post-test period) involved patients hospitalized during 2008 to 2010. During period 1, no difference in the VAP attack rate (number of VAPs per 100 intubated patients) was observed between units A and B (*P *= 0.43). During period 2, the VAP attack rate was higher in unit A compared to unit B (*P *= 0.002). In unit A, the VAP attack rate increased between periods 1 and 2 (*P *<0.001). In unit B, the VAP attack rate did not change between periods 1 and 2 (*P *= 0.17).

The VAP attack rate rose in the intervention group from 7.8% (n = 35) during period 1 to 17.1% (n = 84) during period 2 (*P *<0.001) (Figure 1). The VAP attack rate in the control group without surveillance disruption did not change between periods 1 and 2 (*P *= 0.17).

VAP incidence increased after surveillance disruption in the intervention group (incidence rate ratio (IRR) = 2.17, 95% CI 1.05 to 4.47, *P *= 0.036) independently of VAP trend (Table 2), but remained unchanged in the control group (IRR = 1.37, 95% CI 0.74 to 2.55, *P *= 0.31). Additional file 1 reports the covariables associated with VAP occurrence after univariate analysis. After adjusting for trends, gender, age, patient origin, immunosuppression, diagnosis category, antibiotics and SAPSII at admission, the adjusted incidence of VAP rose after surveillance disruption (IRR = 2.31, 95% CI 1.03 to 5.17, *P *= 0.042), but did not change in the control group (IRR = 1.36, 95% CI 0.72 to 2.56, *P *= 0.35).

**Table 2:** Incidence rate ratio of ventilator-associated pneumonia for trends and disruption in 2007 in ICU

Characteristics	Intervention group: Interrupted surveillance, unit A		Control group: Continuous surveillance, unit B	
		
	**Adjusted IRR of VAP (95% CI)**^a^	*P*	**Adjusted IRR of VAP (95% CI)**^a^	*P*
Bivariate model				
Trend, per quarter	0.98 (0.93 to 1.03)	0.45	1.01 (0.97 to 1.05)	0.69
Period				
Before surveillance disruption^b^	1.00 (Ref.)		1.00 (Ref.)	
After surveillance disruption^c^	2.17 (1.05 to 4.47)	0.036	1.37 (0.74 to 2.55)	0.31
				
Full model^d^				
Trend, per quarter	0.98 (0.92 to 1.04)	0.43	1.02 (0.97 to 1.06)	0.50
Period				
Before surveillance disruption^b^	1.00 (Ref.)		1.00 (Ref.)	
After surveillance disruption^c^	2.31 (1.03 to 5.17)	0.042	1.36 (0.72 to 2.56)	0.35

NOTE: CI, confidence interval; IRR, incidence rate ratio; VAP, ventilator-associated pneumonia. ^a^After multivariate Poisson regression. ^b ^Period 1 (2004 to 2006). ^c ^Period 2 (2007 to 2010). ^d ^Adjusted for gender, age, patient origin, immunosuppression, diagnosis category, antibiotics and SAPSII at admission in the unit. The two other variables, trend (per quarter) and period (period 1 vs. period 2), were forced in the model.

## Discussion

The study objective was to assess the effect of HAI surveillance disruption on VAP incidence in ICUs. We observed increased VAP incidence in the unit with surveillance disruption, independently of time-trends (IRR = 2.17, 95% CI 1.05 to 4.47, *P *= 0.036), and no change in the group without surveillance disruption. The effect we saw could be mainly explained by the lack of data feedback and counselling in unit A during 2007. Indeed, no such outcome was seen in the control group. The effect was robust because the same observation was made after adjusting for gender, age, patient origin, immunosuppression, diagnosis category, antibiotics and SAPSII at admission.

The infection control team would have been less present without surveillance in ICUs, because surveillance needs regular data collection completed by counselling for best practices. In unit A, surveillance was interrupted because of infection control team re-organisation in 2007 and was continuous in 2004 to 2006 and 2008 to 2010. No change in senior physicians occurred in 2007 in unit A. The infection control nurse responsible for HAI surveillance in 2004 to 2006 stopped doing so in 2007 because of overwork, and other infection control practitioners were assumed surveillance in 2008 to 2010. However, VAP definition conformed to a standardised protocol [15,21,22], and no change in the surveillance protocol occurred during the study period. Also, we can be confident that VAP incidence truly increased in unit A after surveillance disruption.

Moreover, during surveillance disruption, the infection control team provided no feedback of HAI surveillance results to the ICU staff. Kasatpibal *et al*. [27] observed no effect of surveillance programme implementation on the incidence of surgical site infection; explanative factors were poor support from hospitals, insufficient enforcement by infection control teams and lack of cooperation with surgeons. On the other hand, a good relationship existed between ICUs and the infection control teams in our hospital, and a previous study reported the impact of standardised preventive measures in all units at risk of HAI (ICUs, haematology units) [28]. The two ICUs investigated were also targeted by these control measures at the same level. Usually, feedback meetings offer good opportunities to discuss prevention practices, such as device utilisation and healthcare organisation. In addition, there are multiple interactions between the infection control department and frontline clinicians other than data feedback. Some of them are: counselling for standard or isolation precautions, investigation of HAI clusters or of HAI caused by multidrug-resistant microorganisms, discussion of specific VAP precautions. These interactions are not formalised but are the cornerstone of a safety culture [29]. They may account for the impact of surveillance upon rates.

Overall mortality, length of stay and length of invasive mechanical ventilation increased after surveillance disruption in unit A. We did not record the causes of death. Also, we could not attribute the excess mortality to increased VAP occurrence. Changes in patient severity or modification of care practices and therapeutics along time could be other explanative factors. However, we did not observe increased mortality or average length of stay in the control unit. It has been suggested that studies on VAP prevention could demonstrate benefits in patient outcome (mortality and duration of stay) [18]. Here, we discerned changes in VAP incidence, mortality and duration of stay in the group with surveillance disruption but not in the control group. Moreover, mean duration of hospital stay in patients who did not suffer from VAP did not change between periods in the group with surveillance disruption; also, the increased incidence of VAP in this unit might have directly impacted global length of stay. In addition, the analysis was adjusted for gender, age, patient origin, immunosuppression, diagnosis category, antibiotics and SAPSII at admission, but residual confounding by severity of illness factors might exist. Moreover, to the best of our knowledge, no significant factors besides the break in surveillance occurred around 2007 or thereafter. There was no new major or expanded surgical or medical programme. VAP prevention recommendations did not change in 2007. The main factors that could explain the increase in VAP rates after disruption of surveillance are lower data feedback and counselling in ICUs. No other intervention occurred in the unit with surveillance disruption as well as in the control unit.

VAP incidence was stable in the ICU with continuous surveillance. In contrast, an inverse relationship was noted in the other ICU: the absence of surveillance correlated with an increased HAI rate. Disruption of active surveillance could be viewed as a lack of "pressure" to control HAI risk in ICUs. During period 2, in response to learning of high VAP rates after surveillance disruption, infection control nurses and practitioners undertook active counselling. Despite this active participation of infection control staff, a residual effect of surveillance disruption was seen as VAP incidence remained higher in the unit for three years thereafter, while global HAI trends were similar in the two units during period 2 (Figure 1).

Reversibility of the surveillance effect reinforces the causal link between HAI surveillance and prevention [30]. Temporality is clearly witnessed here because surveillance disruption precedes the increase in VAP incidence. Other reasons for the causal relationship between surveillance and VAP prevention are the strength of the surveillance disruption effect and plausibility of the preventive outcome of surveillance with feedback to healthcare workers. One could argue that the relationship between surveillance and VAP prevention could be due to an intermediate effect. However, we were unable to detail this intermediate effect more precisely, if it existed.

Our study has some strengths. First, the data were collected prospectively, based on a standardised protocol and VAP definition [15,21,22]. Second, regression analysis permitted us to estimate the effect of surveillance disruption while controlling for time-trends with potential cofounders [26]. Third, the control group allowed us to control the effects of history (events occurring between periods 1 and 2 in addition to the intervention), instrumentation and regression to the mean as well as interaction between these effects [17,19]. Thus, as advanced by Harris *et al*. [19], the presence of a control group reinforced internal validity.

The major limitation of our study is the lack of randomisation of the intervention, which implies that the intervention and control groups are not equivalent [19]. However, HAI surveillance is unit-based, and no individual randomisation is possible. A multicenter study would allow randomisation in clusters, but organisational difficulties limited this perspective. Here, multivariate analysis permitted us to take potential confounding factors into account. However, additional confounding factors, such as chronic co-morbidities (that is, diabetes, renal failure, liver disease, heart disease) or source of immunosuppression (that is, chemotherapy, tumour necrosis factor blockers, steroids), would have been useful but were not collected prospectively. Moreover, accurate VAP diagnosis is difficult, and classification bias of the disease cannot be excluded. However, as no alternative measure of infectious pulmonary complications among ventilated ICU patients currently exists [31], we applied standardised definitions. Moreover, this classification bias might not be related to time. Another point is the monocentric nature of the study that might limit generalisability of the findings. However, this design might reinforce internal validity. Finally, no data on compliance measures associated with VAP prevention (that is, head of bed elevation, sedation vacation) were collected prospectively. However, the protocols related to VAP prevention were similar between the two units. Indeed, infection control protocols were the same in both units because they are standardised in University of Lyon hospitals.

## Conclusions

In summary, we observed an increase in VAP incidence, length of stay and in-hospital mortality after HAI surveillance disruption in ICU. Disruption of surveillance induced lower data feedback and counselling in ICU. This finding suggests a specific effect of HAI surveillance on VAP prevention and reinforces the utility of surveillance systems. HAI surveillance should be considered as a mechanism to facilitate performance improvement and infection control.

Continuous surveillance of HAI incidence in ICUs requires major resources from both infection control units and ICUs. However, it is important to continue to improve this system for very probable direct benefits to patients. Additional data are needed regarding the reasons of stopping of surveillance as potential other preventive measures not done during this period.

Cost-effectiveness analysis is warranted for long-term implementation of HAI surveillance in ICUs.

## Key messages

- The preventive impact of HAI surveillance is not well known.

- It is difficult to perform controlled randomised trials to confirm or refute this premise, and a quasi-experimental design with a control group could be an alternative approach to the question.

- The objective was to investigate the effect of HAI surveillance disruption on VAP incidence.

- VAP incidence, all-cause mortality and length of stay increased in one ICU after surveillance disruption, and no change was observed in the control unit with continuous surveillance.

- HAI surveillance with data feedback and counselling should be considered as a mechanism to facilitate performance improvement and infection control in ICUs.

## Abbreviations

BAL: broncho-alveolar lavage; CI: confidence interval; HAI: hospital-acquired infection; ICU: intensive care unit; IRR: incidence rate ratio; SAPSII: simplified acute physiological score II; SD: standard deviation; VAP: ventilator-associated pneumonia

## Competing interests

The authors declare that they have no competing interests.

## Authors' contributions

TB participated in study conception and design, performed statistical analysis and drafted the initial manuscript. BA and LA participated in data analysis and interpretation and helped to draft the manuscript. PV conceived the study, participated in data analysis and interpretation and helped to draft the manuscript. All authors read and approved the final manuscript.

## Supplementary Material

Additional file 1 **Additional Table **1. Covariables associated with ventilator associated pneumonia incidence in unit A (interrupted VAP surveillance) vs. unit B (continuous VAP surveillance), Edouard Herriot Hospital, 2004 to 2010 - univariate Poisson regression. CI, confidence interval; IRR, incidence rate ratio; SAPSII, simplified acute physiological score II; VAP, ventilator-associated pneumonia. Click here for file
