# Supplementary material for: Impact of surveillance of hospital-acquired infections on the incidence of ventilator-associated pneumonia in intensive care units: a quasi-experimental study
Source: Crit Care. 2012 Aug 21;16(4):R161. doi: 10.1186/cc11484 (PMC3580751; doi:10.1186/cc11484)
Supplement: Additional file 1 — Additional Table 1. Covariables associated with ventilator associated pneumonia incidence in unit A (interrupted VAP surveillance) vs. unit B (continuous VAP surveillance), Edouard Herriot Hospital, 2004 to 2010 - univariate Poisson regression. CI, confidence interval; IRR, incidence rate ratio; SAPSII, simplified acute physiological score II; VAP, ventilator-associated pneumonia. [file cc11484-S1.PDF]

Additional file 1.

Supplementaty Table 1. Covariables associated with ventilator associated pneumonia incidence in unit A (interrupted VAP surveillance) vs. unit B (continuous VAP surveillance), Edouard Herriot Hospital, 2004-2010 – univariate Poisson regression

Characteristics	Intervention group: Interrupted surveillance, unit A		Control group: Continuous surveillance, unit B	
	Crude IRR of VAP (95% CI)	*P*	Crude IRR of VAP (95% CI)	*P*
Gender, male	1.22 (0.82-1.81)	0.33	1.08 (0.81-1.46)	0.60
Age at admission				
<50 years	1.00 (Ref.)		1.00 (Ref.)	
50-69 years	1.07 (0.66-1.73)	0.79	1.32 (0.89-1.97)	0.17
≥70 years	0.85 (0.50-1.47)	0.57	1.07 (0.71-1.61)	0.76
Patient origin				
Home	1.00 (Ref.)		1.00 (Ref.)	
Other unit/hospital	0.65 (0.43-0.98)	0.04	0.74 (0.56-1.00)	0.04
Immunosuppressed	0.89 (0.62-1.28)	0.53	0.77 (0.52-1.14)	0.19
Diagnosis category				
Medical	1.00 (Ref.)		1.00 (Ref.)	
Surgery	1.11 (0.77-1.59)	0.57	1.74 (1.12-2.69)	0.013
Antibiotics at admission	0.67 (0.46-0.97)	0.033	0.87 (0.64-1.20)	0.41
SAPSII				
<40	1.00 (Ref.)		1.00 (Ref.)	
40-59	1.13 (0.71-1.79)	0.60	1.07 (0.72-1.59)	0.73
≥60	1.11 (0.70-1.77)	0.66	0.92 (0.62-1.37)	0.69

NOTE: CI, confidence interval, IRR, incidence rate ratio; SAPSII**,** simplified acute physiological score II; VAP, ventilator-associated pneumonia
